# A multi-site, multi-disorder resting-state magnetic resonance image database

**DOI:** 10.1038/s41597-021-01004-8

**Published:** 2021-08-30

**Authors:** Saori C. Tanaka, Ayumu Yamashita, Noriaki Yahata, Takashi Itahashi, Giuseppe Lisi, Takashi Yamada, Naho Ichikawa, Masahiro Takamura, Yujiro Yoshihara, Akira Kunimatsu, Naohiro Okada, Ryuichiro Hashimoto, Go Okada, Yuki Sakai, Jun Morimoto, Jin Narumoto, Yasuhiro Shimada, Hiroaki Mano, Wako Yoshida, Ben Seymour, Takeshi Shimizu, Koichi Hosomi, Youichi Saitoh, Kiyoto Kasai, Nobumasa Kato, Hidehiko Takahashi, Yasumasa Okamoto, Okito Yamashita, Mitsuo Kawato, Hiroshi Imamizu

**Affiliations:** 1 Brain Information Communication Research Laboratory Group, Advanced Telecommunications Research Institutes International, Kyoto, Japan; 2 Department of Psychiatry, Boston University School of Medicine, Massachusetts, USA; 3 Department of Neuropsychiatry, Graduate School of Medicine, The University of Tokyo, Tokyo, Japan; 4 Institute for Quantum Life Science, National Institutes for Quantum and Radiological Science and Technology, Chiba, Japan; 5 Medical Institute of Developmental Disabilities Research, Showa University, Tokyo, Japan; 6 Brain, Mind and KANSEI Sciences Research Center, Hiroshima University, Hiroshima, Japan; 7 Department of Psychiatry, Kyoto University Graduate School of Medicine, Kyoto, Japan; 8 Department of Radiology, IMSUT Hospital, Institute of Medical Science, The University of Tokyo, Tokyo, Japan; 9 Department of Radiology, Graduate School of Medicine, The University of Tokyo, Tokyo, Japan; 10 The International Research Center for Neurointelligence (WPI-IRCN) at the University of Tokyo Institutes for Advanced Study (UTIAS), Tokyo, Japan; 11 Department of Language Sciences, Tokyo Metropolitan University, Tokyo, Japan; 12 Department of Psychiatry and Neurosciences, Graduate School of Biomedical and Health Sciences, Hiroshima University, Hiroshima, Japan; 13 Department of Psychiatry, Graduate School of Medical Science, Kyoto Prefectural University of Medicine, Kyoto, Japan; 14 Brain Activity Imaging Center, ATR-Promotions Inc., Kyoto, Japan; 15 Center for Information and Neural Networks (CiNet), National Institute of Information and Communications Technology (NICT), Osaka, Japan; 16 Laboratory of Single Molecule Imaging, WPI Immunology Frontier Research Center, Osaka University, Osaka, Japan; 17 Department of Systems Science, Graduate School of Informatics, Kyoto University, Kyoto, Japan; 18 The Wellcome Centre for Integrative Neuroimaging, University of Oxford, Oxford, UK; 19 Department of Neuromodulation and Neurosurgery, Osaka University Graduate School of Medicine, Osaka, Japan; 20 Department of Neurosurgery, Osaka University Graduate School of Medicine, Osaka, Japan; 21 Department of Psychiatry and Behavioral Sciences, Tokyo Medical and Dental University, Tokyo, Japan; 22 Center for Advanced Intelligence Project, RIKEN, Tokyo, Japan; 23 Department of Psychology, Graduate School of Humanities and Sociology, The University of Tokyo, Tokyo, Japan

**Keywords:** Neural circuits, Diagnostic markers, Psychiatric disorders, Neurological disorders

## Abstract

Machine learning classifiers for psychiatric disorders using resting-state functional magnetic resonance imaging (rs-fMRI) have recently attracted attention as a method for directly examining relationships between neural circuits and psychiatric disorders. To develop accurate and generalizable classifiers, we compiled a large-scale, multi-site, multi-disorder neuroimaging database. The database comprises resting-state fMRI and structural images of the brain from 993 patients and 1,421 healthy individuals, as well as demographic information such as age, sex, and clinical rating scales. To harmonize the multi-site data, nine healthy participants (“traveling subjects”) visited the sites from which the above datasets were obtained and underwent neuroimaging with 12 scanners. All participants consented to having their data shared and analyzed at multiple medical and research institutions participating in the project, and 706 patients and 1,122 healthy individuals consented to having their data disclosed. Finally, we have published four datasets: 1) the SRPBS Multi-disorder Connectivity Dataset 2), the SRPBS Multi-disorder MRI Dataset (restricted), 3) the SRPBS Multi-disorder MRI Dataset (unrestricted), and 4) the SRPBS Traveling Subject MRI Dataset.

## Abstract (summary)

**Table:** 

Measurement(s)	mental or behavioural disorder • brain measurement • Demographic Data
Technology Type(s)	functional magnetic resonance imaging • magnetic resonance imaging • Resting State Functional Connectivity Magnetic Resonance Imaging
Factor Type(s)	age • sex • site • disorder
Sample Characteristic - Organism	Homo sapiens

Machine-accessible metadata file describing the reported data: 10.6084/m9.figshare.14716329

## Background & Summary

Diagnostic criteria based on psychiatric symptoms, such as the Diagnostic and Statistical Manual of Mental Disorders (DSM)^1^ and the International Classification of Diseases (ICD)^2^, are commonly used in clinical practice and research. However, overlapping and heterogeneous clinical presentations of psychiatric disorders have compromised their validity^3–6^. It has been suggested that a new framework enabling objective diagnosis is needed for research to properly interpret the etiology and pathophysiology of psychiatric disorders and to treat them effectively. To address this issue, the Research Domain Criteria initiative of the U.S. National Institute of Mental Health has analyzed the genes, molecules, cells, neurophysiology, and behavior involved in functional domains associated with specific neural circuits, with the aim of illuminating the biological basis of mental disorders^7^.

As a method for directly examining the relationship between neural circuits and psychiatric disorders, machine learning classifiers using resting-state functional magnetic resonance imaging (rs-fMRI) have recently attracted attention. Functional connectivity (i.e., the degree to which activity patterns are synchronized between particular brain regions, as revealed with rs-fMRI) is associated with a variety of individual characteristics, as well as psychiatric disorders^8–12^. Connectivity-based classifiers have been developed for many psychiatric disorders, including autism spectrum disorder^13^, depression^14^, schizophrenia^15^, and obsessive-compulsive disorder^16^.

For development of accurate classifiers, large-scale datasets are necessary. In particular, generalizable classifiers that can be replicated with data collected at independent sites require data collection from multiple sites. In multi-site data collection, it is necessary to develop a method for canceling out differences between sites and integrating them into a homogeneous dataset, a process known as data harmonization^17^. Multi-site, multi-disorder data collection is also important to investigate the full spectrum of diseases and disease subtypes. In this context, the development of a harmonizable database is an urgent issue.

To address this issue, the DecNef Consortium (https://bicr.atr.jp/decnefpro/) introduced unified data collection protocols covering multiple sites and disorders. In 2013, around 60 neuroscientists, neuropsychiatrists, engineers, computational scientists, and psychologists from eight Japanese universities and institutes formed the consortium as part of the Japanese Strategic Research Program for the Promotion of Brain Science (SRPBS) project for big data applications, machine-learning algorithms, and sophisticated fMRI neurofeedback methods for diagnosing and treating multiple psychiatric disorders. Many of these techniques are related to brain-machine interfaces. SRPBS, a nation-wide research program for brain science, is supported by the Japanese Advanced Research and Development Programs for Medical Innovation (AMED).

Neuroimaging data of 2,414 patients and control participants were collected at eight sites (Table 1). A coherent protocol was designed and used for rs-fMRI (see MRI acquisition in Methods). Table 1 shows the number of samples collected for each disorder at each site. Altogether, 14 scanners from three manufacturers (Siemens, Philips, and GE) were used to produce these neuroimaging data.

**Table 1:** Summary of the SRPBS database project.

Institute	MRI scanner	Autism spectrum disorder	Major depressive disorder/Bipolar	Obsessive compulsive disorder	Schizophrenia spectrum disorders	Chronic pain/Back pain	Others	Healthy and typically developed controls	Total
University of Tokyo, Tokyo	Philips, GE	10	103	—	36	—	32	170	351
Osaka University, Osaka	Siemens	—	—	—	—	43	10	29	82
Showa University, Tokyo	Siemens	115	—	—	19	—	—	110	244
Kyoto University, Kyoto	Siemens	—	25	—	104			244	373
Hiroshima University, Hiroshima	Siemens, GE	—	318	—	—	40	—	468	826
Advanced Telecommunications Research Institute (ATR), Kyoto	Siemens	—	4	5	—	—	—	271	280
Kyoto Prefectural University of Medicine, Kyoto	Philips	—	—	105	—	—	—	90	195
CiNet, Osaka	Siemens	—	—	—	—	24	—	39	63
Total	—	125	450	110	159	107	42	1421	2414

To collect a large amount of neuroimaging data associated with psychiatric disorders, images must be acquired from multiple sites because of the limited capacity of a single site. To properly manage heterogeneous, multisite data, it is important to understand what leads to differences in data across sites and to develop a method for harmonizing the data. To this end, 143 sessions were acquired from 9 traveling subjects who visited the consortium sites (12 scanners). Using the traveling-subject dataset in conjunction with the multi-disorder dataset, we demonstrated that site differences are due to biological sampling bias (differences between participant groups) and engineering measurement bias (differences in the properties of the MRI scanners used)^17^.

We have published four datasets generated from the SRPBS database for different purposes:

1. The SRPBS Multi-disorder Connectivity Dataset consists of rs-fMRI data from patients and control participants^18^
2. The SRPBS Multi-disorder MRI Dataset (restricted) consists of rs-fMRI and structural MRI images of patients and control participants^19^
3. The SRPBS Multi-disorder MRI Dataset (unrestricted) consists of (resting-state) functional and structural MRI images of patients and participants^20^
4. The SRPBS Traveling Subject MRI Dataset consists of (resting-state) functional and structural MRI images of traveling participants^21^

**Fig. 1:**
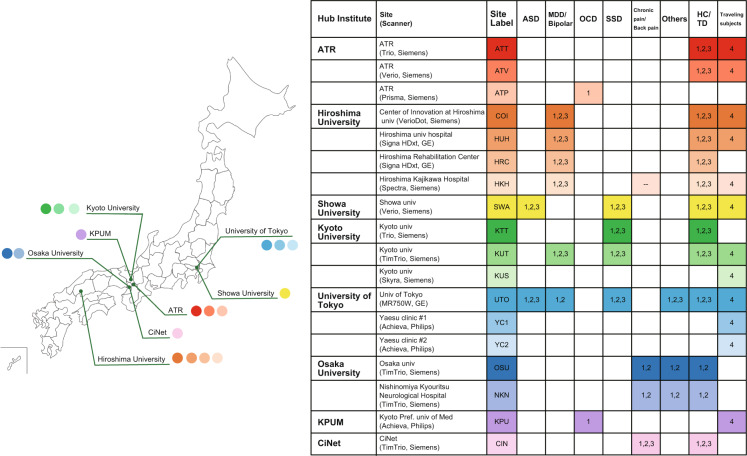
Summary of the sites and datasets. The map of Japan shows the location of each hub institute involved in data collection. Each color dot on the map indicates the site (scanner) listed in the legend table, which lists the sites and disorders included in Datasets 1–4. Numbers in the table indicate the dataset number. ASD, autism spectrum disorder; MDD, major depressive disorder; OCD, obsessive compulsive disorder; SSD, schizophrenia spectrum disorders; HC, healthy controls; TD, typically developed; KPUM, Kyoto Prefectural University of Medicine; CiNet, Center for Information and Neural Networks.

Sites and datasets are summarized in Fig. 1.

## Methods

### Ethics statement

All participants in all datasets provided written informed consent. All recruitment procedures and experimental protocols were approved by the institutional review boards of the principal investigators’ respective institutions (Advanced Telecommunications Research Institute International [approval numbers: 13–133, 14–133, 15–133, 16–133, 17–133, and 18–133], Hiroshima University [E-172, E-38], Kyoto Prefectural University of Medicine [RBMR-C-1098], Showa University [B-2014-019 and UMIN000016134], the University of Tokyo Faculty of Medicine [3150], Kyoto University [C809 and R0027], Osaka University [13384], and CiNet [20140611]). This study was conducted in accordance with the Declaration of Helsinki.

### Participants

Demographic characteristics of the participants included in the SRPBS Multi-disorder Connectivity Dataset (Table 2), the SRPBS Multi-disorder MRI Dataset (Table 3), and the OPEN SRPBS Multi-disorder MRI dataset (Table 4) are summarized below.

**Table 2:** Demographic characteristics of participants included in the SRPBS Multi-disorder Connectivity Dataset.

Site		HC	ASD	MDD	OCD	Schizophrenia	Pain	Stroke	Others	Bipolar	Dysthymia	Schizoaffective	All
SWA	No., M:W	101, 86:15	115, 100:15	—	—	19, 15:4	—	—	—	—	—	—	235, 201:34
	Age, y	28.4 (7.9)	32.1 (7.8)	—	—	42.9 (8.4)	—	—	—	—	—	—	31.4 (8.7)
HUH	No., M:W	63, 29:34	—	57, 32:25	—	—	—	—	—	—	—	—	120, 61:59
	Age, y	34.3 (13.2)	—	43.3 (12.2)	—	—	—	—	—	—	—	—	38.6 (13.4)
HRC	No., M:W	49, 13:36	—	15, 5:10	—	—	—	—	—	—	—	—	64, 18:46
	Age, y	41.7 (11.7)	—	38.6 (8.9)	—	—	—	—	—	—	—	—	41.0 (11.1)
HKH	No., M:W	29, 12:17	—	33, 20:13	—	—	—	—	—	—	—	—	62, 32:30
	Age, y	45.4 (9.5)	—	44.8 (11.5)	—	—	—	—	—	—	—	—	45.1 (10.5)
COI	No., M:W	120, 43:77	—	68, 28:40	—	—	—	—	—	—	—	—	188, 71:117
	Age, y	51.7 (13.3)	—	44.8 (12.5)									49.2 (13.4)
KUT	No., M:W	159, 93:66	—	16, 10:6	—	44, 20:24	—	—	—	—	—	1, 1:0	220, 124:96
	Age, y	36.5 (13.6)	—	42.6 (12.5)		41.3 (10.9)							38.0 (13.1)
KTT	No., M:W	75, 48:27	—	—	—	44, 25:19	—	—	—	—	—	3, 1:2	122, 74:48
	Age, y	28.9 (9.1)	—	—	—	37.3 (9.7)	—	—	—	—	—		32.4 (10.3)
UTO	No., M:W	170, 78:92	10, 9:1	62, 36:26	—	35, 23:12	—	—	28, 15:13	41, 26:15	4, 3:1	—	350, 190:160
	Age, y	35.6 (17.5)	37.0 (9.6)	38.7 (11.6)	—	31.7 (10.4)	—	—	33.5 (13.0)	34.2 (9.1)	30.3 (16.2)	—	35.4 (14.6)
ATT	No., M:W	31, 28:3	—	—	—	—	—	—	—	—	—	—	31, 28:3
	Age, y	23.0 (1.9)	—	—	—	—	—	—	—	—	—	—	23.0 (1.9)
ATV	No., M:W	77, 60:17	—	—	—	—	—	—	—	—	—	—	77, 60:17
	Age, y	22.7 (2.0)	—	—	—	—	—	—	—	—	—	—	22.7 (2.0)
ATP	No., M:W	—	—	—	2, 1:1	—	—	—	—	—	—	—	2, 1:1
	Age, y	—	—	—	33.5 (16.3)	—	—	—	—	—	—	—	33.5 (16.3)
KPU	No., M:W	—	—	—	9, 4:5	—	—	—	—	—	—	—	9, 4:5
	Age, y	—	—	—	30.1 (9.1)	—	—	—	—	—	—	—	30.1 (9.1)
OSU	No., M:W	27, 19:8	—	—	—	—	37, 24:14	9, 6:3	—	—	—	—	73, 49:24
	Age, y	61.2 (11.8)	—	—	—	—	61.1 (9.8)	67.7 (5.7)	—	—	—	—	61.9 (10.3)
NKN	No., M:W	2, 2:0	—	—	—	—	6, 3:3	1, 0:1	—	—	—	—	9, 5:4
	Age, y	59.5 (0.7)	—	—	—	—	64.2 (23.2)	68.0 (0.0)	—	—	—	—	63.6 (18.5)
CIN	No., M:W	39, 25:14	—	—	—	—	24, 12:12	—	—	—	—	—	63, 37:26
	Age, y	38.7 (13.5)	—	—	—	—	45.5 (11.1)	—	—	—	—	—	41.3 (13.0)
Summary	No., M:W	942, 536:406	125, 109:16	251, 131:120	11, 5:6	142, 83:59	67, 39:28	10, 6:4	28, 5:13	41, 26:15	4, 3:1	4, 2:2	1625, 955:670
	Age, y	36.5 (15.5)	32.5 (8.0)	42.5 (12.1)	30.7 (9.8)	37.9 (10.8)	55.8 (14.0)	67.7 (5.4)	33.5 (13.0)	34.2 (9.1)	30.3 (16.2)	46.8 (5.7)	38.1 (14.7)

Data are shown as means (standard deviation). M, men; W, women; HC, healthy controls; ASD, autism spectrum disorder; MDD, major depressive disorder; OCD, obsessive compulsive disorder; SWA, Showa university; HUH, Hiroshima University Hospital; HRC, Hiroshima Rehabilitation Center; HKH, Hiroshima Kajikawa Hospital; COI, Hiroshima COI; KUT, Kyoto University TimTrio; KTT, Kyoto University Trio; UTO, University of Tokyo Hospital; ATT, ATR Trio; ATV, ATR Verio; ATP, ATR Prisma; CIN, CiNet; NKN, Nishinomiya Kyouritsu Hospital; KPU, Kyoto Prefectural University of Medicine.

**Table 3:** Demographic characteristics of participants included in the SRPBS Multi-disorder MRI Dataset (restricted).

Site		HC	ASD	MDD	OCD	SSD	Pain	Stroke	Bipolar	Dysthymia	Others	All
SWA	No., M:W	101, 86:15	115, 100:15	—	—	19, 15:4	—	—	—	—	—	235, 201:34
	Age, y	28.4 (7.9)	32.1 (7.8)	—	—	42.9 (8.4)	—	—	—	—	—	31.4 (8.7)
HUH	No., M:W	67, 29:38	—	57, 32:25	—	—	—	—	—	—	—	124, 61:63
	Age, y	34.7 (13.0)	—	43.3 (12.2)	—	—	—	—	—	—	—	38.7 (13.3)
HRC	No., M:W	49, 13:36	—	16, 6:10	—	—	—	—	—	—	—	65, 19:46
	Age, y	41.7 (11.7)	—	40.5 (11.5)	—	—	—	—	—	—	—	41.4 (11.5)
HKH	No., M:W	29, 12:17	—	33, 20:13	—	—	—	—	—	—	—	62, 32:30
	Age, y	45.4 (9.5)	—	44.8 (11.5)	—	—	—	—	—	—	—	45.1 (10.5)
COI	No., M:W	124, 46:78	—	71, 31:40	—	—	—	—	—	—	—	195, 77:118
	Age, y	51.9 (13.4)	—	45.2 (12.5)	—	—	—	—	—	—	—	49.4 (13.5)
KUT	No., M:W	159, 93:66	—	16, 10:6	—	45, 21:24	—	—	—	—	—	220, 124:96
	Age, y	36.5 (13.6)	—	42.6 (12.5)	—	41.4 (10.8)	—	—	—	—	—	38.0 (13.1)
KTT	No., M:W	75, 48:27	—	—	—	47, 26:21	—	—	—	—	—	122, 74:48
	Age, y	28.9 (9.1)	—	—	—	37.9 (9.8)	—	—	—	—	—	32.4 (10.3)
UTO	No., M:W	170, 78:92	10, 9:1	62, 36:26	—	36, 24:12	—	—	41, 26:15	4, 3:1	28, 15:13	351, 191:160
	Age, y	35.6 (17.5)	37.0 (9.6)	38.7 (11.6)	—	31.4 (10.3)	—	—	34.2 (9.1)	30.3 (16.2)	33.5 (13.0)	35.4 (14.6)
ATT	No., M:W	31, 28:3	—	—	—	—	—	—	—	—	—	31, 28:3
	Age, y	23.0 (1.9)	—	—	—	—	—	—	—	—	—	23.0 (1.9)
ATV	No., M:W	77, 60:17	—	—	—	—	—	—	—	—	—	77, 60:17
	Age, y	22.7 (2.0)	—	—	—	—	—	—	—	—	—	22.7 (2.0)
OSU	No., M:W	27, 19:8	—	—	—	—	37, 24:13	9, 6:3	—	—	—	73, 49:24
	Age, y	61.2 (11.8)	—	—	—	—	61.1 (9.8)	67.7 (5.7)	—	—	—	61.9 (10.3)
CIN	No., M:W	39, 25:14	—	—	—	—	24, 12:12	—	—	—	—	63, 37:26
	Age, y	38.7 (13.5)	—	—	—	—	45.5 (11.1)	—	—	—	—	41.3 (13.0)
NKN	No., M:W	2, 2:0	—	—	—	—	6, 3:3	1, 0:1	—	—	—	9, 5:4
	Age, y	59.5 (0.7)	—	—	—	—	64.2 (23.2)	68.0 (0.0)	—	—	—	63.6 (18.5)
Summary	No., M:W	950, 539:411	125, 135:120	255, 135:120	—	147, 86:61	67, 39:28	10, 6:4	41, 26:15	4, 3:1	28, 15:13	1627, 958:669
	Age, y	36.6 (15.5)	42.7 (12.2)	42.7 (12.2)	—	38.0 (10.8)	55.8 (14.0)	67.7 (5.4)	34.2 (9.1)	30.3 (16.2)	33.5 (13.0)	38.2 (14.8)

Data are shown as means (standard deviation). M, men; W, women; HC, healthy controls; ASD, autism spectrum disorder; MDD, major depressive disorder; OCD, obsessive compulsive disorder; SSD, schizophrenia spectrum disorders; SWA, Showa university; HUH, Hiroshima University Hospital; HRC, Hiroshima Rehabilitation Center; HKH, Hiroshima Kajikawa Hospital; COI, Hiroshima COI; KUT, Kyoto University TimTrio; KTT, Kyoto University Trio; UTO, University of Tokyo Hospital; ATT, ATR Trio; ATV, ATR Verio; OSU, Osaka university; CIN, CiNet; NKN, Nishinomiya Kyouritsu Hospital.

**Table 4:** Demographic characteristics of participants included in the SRPBS Multi-disorder MRI Dataset (unrestricted).

Site		HC	ASD	MDD	OCD	SSD	Pain	Stroke	Bipolar	Dysthymia	Others	All
SWA	No., M:W	101, 86:15	115, 100:15	—	—	19, 15:4	—	—	—	—	—	235, 201:34
	Age, y	28.4 (7.9)	32.1 (7.8)	—	—	42.9 (8.4)	—	—	—	—	—	31.4 (8.7)
HUH	No., M:W	67, 29:38	—	57, 32:25	—	—	—	—	—	—	—	124, 61:63
	Age, y	34.7 (13.0)	—	43.3 (12.2)	—	—	—	—	—	—	—	38.7 (13.3)
HRC	No., M:W	49, 13:36	—	16, 6:10	—	—	—	—	—	—	—	65, 19:46
	Age, y	41.7 (11.7)	—	40.5 (11.5)	—	—	—	—	—	—	—	41.4 (11.5)
HKH	No., M:W	29, 12:17	—	33, 20:13	—	—	—	—	—	—	—	62, 32:30
	Age, y	45.4 (9.5)	—	44.8 (11.5)	—	—	—	—	—	—	—	45.1 (10.5)
COI	No., M:W	124, 46:78	—	71, 31:40	—	—	—	—	—	—	—	195, 77:118
	Age, y	51.9 (13.4)	—	45.2 (12.5)	—	—	—	—	—	—	—	49.4 (13.5)
KUT	No., M:W	159, 93:66	—	16, 10:6	—	45, 21:24	—	—	—	—	—	220, 124:96
	Age, y	36.5 (13.6)	—	42.6 (12.5)	—	41.4 (10.8)	—	—	—	—	—	38.0 (13.1)
KTT	No., M:W	75, 48:27	—	—	—	47, 26:21	—	—	—	—	—	122, 74:48
	Age, y	28.9 (9.1)	—	—	—	37.9 (9.8)	—	—	—	—	—	32.4 (10.3)
UTO	No., M:W	96, 33:63	10, 9:1	62, 36:26	—	36, 24:12	—	—	41, 26:15	4, 3:1	23, 11:12	272, 142:130
	Age, y	47.0 (15.5)	37.0 (9.6)	38.7 (11.6)	—	31.4 (10.3)	—	—	34.2 (9.1)	30.3 (16.2)	36.3 (12.7)	39.6 (14.0)
ATT	No., M:W	13, 12:1	—	—	—	—	—	—	—	—	—	13, 12:1
	Age, y	22.2 (1.4)	—	—	—	—	—	—	—	—	—	22.2 (1.4)
ATV	No., M:W	39 (29:10)	—	—	—	—	—	—	—	—	—	39, 29:10
	Age, y	22.7 (2.2)	—	—	—	—	—	—	—	—	—	22.7 (2.2)
CIN	No., M:W	39, 25:14	—	—	—	—	24, 12:12	—	—	—	—	63, 37:26
	Age, y	38.7 (13.5)	—	—	—	—	45.5 (11.1)	—	—	—	—	41.3 (13.0)
Summary	No., M:W	791, 426:365	125, 109:16	255, 135:120	—	147, 86:61	24, 12:12	—	41, 26:15	4, 3:1	23, 11:12	1410, 808:602
	Age, y	18.1 (15.0)	32.5 (8.0)	42.7 (12.2)	—	38.0 (10.8)	45.5 (11.1)	—	34.2 (9.1)	30.3 (16.2)	36.3 (12.7)	38.4 (13.6)

Data are shown as means (standard deviation). M, men; W, women; HC, healthy controls; ASD, autism spectrum disorder; MDD, major depressive disorder; OCD, obsessive compulsive disorder; SSD, schizophrenia spectrum disorders; SWA, Showa university; HUH, Hiroshima University Hospital; HRC, Hiroshima Rehabilitation Center; HKH, Hiroshima Kajikawa Hospital; COI, Hiroshima COI; KUT, Kyoto University TimTrio; KTT, Kyoto University Trio; UTO, University of Tokyo Hospital; ATT, ATR Trio; ATV, ATR Verio; CIN, CiNet; NKN, Nishinomiya Kyouritsu Hospital.

Participants in Datasets 2 and 3 were included in Dataset 1 and consented to having their MRI data disclosed after anonymization. Dataset 1 participants who were not included in Datasets 2 and 3 consented to having their calculated functional connectivity disclosed, but not their MRI data. Dataset 3 includes participants who consented to having their data shared publicly. The demographic characteristics of participants and the metrics for assessing the quality of the MRI data for each dataset are summarized below.

In Dataset 4 (the SRPBS Traveling Subject dataset), 9 healthy participants (all male; age range 24–32 y; mean age 27 ± 2.6 y) underwent scanning at each of the 12 scanners across the 8 SRPBS consortium sites, producing a total of 143 scan sessions.

### Clinical assessments

Patients with psychiatric/neurological disorders were diagnosed at each site as follows.

#### Hiroshima University

An expert clinician made diagnoses according to the Diagnostic and Statistical Manual of Mental Disorders, Fourth Edition (text revision) (DSM-IV-TR) or Fifth Edition (DSM-5). To confirm the diagnosis, the Mini-International Neuropsychiatric Interview (MINI)^22^ was conducted at the time of participation in the study.

#### Showa University

For patients with autism spectrum disorder, a clinical team assessed the developmental history, present illness, life history, and family history and then made clinical diagnoses according to DSM-IV-TR. For patients with schizophrenia, diagnoses were made by 2 experienced psychiatrists, based on the Structured Clinical Interview for DSM-IV Axis I Disorders-Patient Edition (SCID). Typically developed controls were confirmed to have no psychiatric conditions, according to the Japanese version of the MINI.

#### University of Tokyo

Psychiatric disorders were diagnosed according to DSM-IV criteria. The MINI was used to screen healthy controls for psychiatric disorders.

#### Osaka University and CiNet

Chronic pain was diagnosed based on the definition by the International Association for the Study of Pain: “pain that extends beyond the expected period of healing or progressive pain due to non-cancer diseases^23^.”

#### Kyoto University

Schizophrenia spectrum disorder, major depressive disorder/bipolar disorder, and healthy controls were diagnosed using the SCID.

#### KPUM

Patients with obsessive-compulsive disorder (OCD) were primarily diagnosed using the SCID. Experienced clinical psychiatrists or psychologists applied the Yale–Brown Obsessive-Compulsive Scale^24^ for clinical evaluation of obsessive-compulsive symptoms in patients with OCD.

### MRI acquisition

The SRPBS MRI guideline recommended that MRI data be acquired using the following imaging protocol.

1) rs-fMRI

Scan the entire brain, including the cerebellum, and minimize the repetition time (TR). Emphasize the prefrontal regions that are related to psychiatric disorders.

- Coil: 8/12 ch phased array coil (24/32 ch coil is also acceptable)
- Sequence: ep2d_bold (Siemens)

        –      Please find the corresponding parameters for other vendors.

- Series: T2*-weighted image
- No motion correction
- No SENSE (GRAPPA)
- TR: 2.5 s
- TE: 30 ms
- Flip angle: 80 deg
- Phase encoding: PA
- Matrix: 64 × 64
- FOV: 212 mm
- In-plane resolution: 3.3 × 3.3 mm

        –      Reason: we sometimes find that prefrontal regions are elongated and included in the field of view (FoV) when phase encoding is set at PA and in-plane resolution is set at 3 × 3 mm.

- Slice thickness: 3.2 mm
- Gap: 0.8 mm (25% of slice thickness)

        –      We recommend setting (Slice Thickness + Gap) at an integer value (4) to prevent reading error of the statistical parametric mapping (SPM). Siemens users can set a slice gap as an integer value corresponding to the percentage of slice thickness: gap = slice thickness (3.2 mm) × 25% = 0.8 mm.

- Number of slices: 40 (ideal value)

        –      Maintain the TR at 2.5 s. The ideal number of slices is 40. Slice positions should be set so that there is a considerable margin between the top slice and the top of the head. It is acceptable for the bottom of the cerebellum to stick out of the FoV if the entire brain cannot be covered by the FoV.

- Trans-axial

        –      In principle, we do not recommend oblique slices, but it is acceptable to optimize slice angles if required for a specific research purpose or scanner properties.

- Ascending acquisition (suitable for connectivity analysis)
- Bandwidth: minimum (1736–2500 Hz/px)

        –      You can increase the number of slices by increasing this value when there is a decrease in scanning time. However, scanners may alert you to increase this value. You may ignore the alert, depending on your institution’s policy regarding the use of scanning modes other than the default setting. If you can use only the standard mode, the number of slices should be reduced.

- Number of scanning volume: 240 volumes + 4 dummy volumes

        –      Four dummy volumes at the beginning of each run

- Scanning time: 10 min. + 10 s (dummy)
- Fat suppression: ON
- Acquisition time: Equidistant (=TR-TR/(Number of slices))
  2) B0 field map
  Field mapping is necessary to correct distortion in the prefrontal regions (about 2 min.)
- Sequence: gre_field_mapping (Siemens)

        –      Please find the corresponding parameters for other vendors (2D multi-slice is also acceptable)

- Series: Field mapping
- Reconstruction (phase + magnitude)
- TR: 488 ms (Siemens)
- Flip angle: 60 deg.
- TE: 4.92 ms, 7.38 ms
- Phase encoding: PA
- FOV: 212 mm
- Matrix: 64 × 64
- In-plane resolution: 3.3 × 3.3 mm
- Slice thickness: 3.2 mm
- Gap: 0.8 mm
- Ascending acquisition
- Fat suppression: OFF
- Trans-axial
- Number of slices: 40 (aligned to EPI)
- Bandwidth: minimum (about 260 Hz/px)

        –      Minimize this value as much as possible if it cannot be set at 260 Hz.

- Phase encoding: fat shift (L)
  3) Structure image
- Parameters for structure images conform to those of J-ADNI2 (high-speed mode: GRAPPA/No SENSE)
- 1 mm × 1 mm × 1 mm isovoxel (J-ADNI2 1 mm × 1 mm × 1.2 mm)
- No specification of slice numbers (in the right-to-left direction), but please include the entire brain with a considerable margin.
  4) Instructions to participants and others
- Instructions

        –      Please relax and look at the fixation point.
        –      Do not sleep.
        –      Do not think about anything in particular.
        –      Do not move your body, especially your head and trunk.

- Display Image

        –      A black cross within the fovea is displayed as a fixation point with a gray background that minimizes visual stimulation.

- Environment

        –      Carefully fix the participant’s head and trunk.
        –      The room should be dimly lit.
        –      Please use natural beige UF foam ear plugs (NRR 32).
        –      Please place headphone-style earmuffs over the earplugs.
        –      It is advisable to monitor heart rate and respiration rate if possible.

- Debriefing

        –      Evaluate sleepiness on the Stanford Sleepiness Scale.
        –      Ask participants to confirm that they followed the instructions (we prepared a unified questionnaire).

Multi-disorder data for Datasets 1–3 were acquired at SRPBS consortium sites. Each participant underwent a single rs-fMRI session, a structural MRI session, and an optional field-map session. Detailed imaging parameters used at each site for fMRI and T1-weighted (T1w) structural MRI are summarized in Tables 5 and 6, respectively.

**Table 5:** Imaging protocols for rs-fMRI in the SRPBS Multi-disorder Connectivity and MRI Datasets.

Site	SWA	HUH	HRC	HKH	COI	KUT	KTT	UTO	ATV	ATV	ATT	ATT	KPU	OSU, NKN	CIN
MRI scanner	*Siemens*	*GE*	*GE*	*Siemens*	*Siemens*	*Siemens*	*Siemens*	*GE*	*Siemens*	*Siemens*	*Siemens*	*Siemens*	*Philips*	*Siemens*	*Siemens*
	*Verio*	*Signa HDxt*	*Signa HDxt*	*Spectra*	*Verio.Dot*	*TimTrio*	*Trio*	*MR750w*	*Verio*	*Verio*	*TimTrio*	*TimTrio*	*Achieva*	*TimTrio*	*TimTrio*
Magnetic field strength	3.0 T	3.0 T	3.0 T	3.0 T	3.0 T	3.0 T	3.0 T	3.0 T	3.0 T	3.0 T	3.0 T	3.0 T	3.0 T	3.0 T	3.0 T
No.of channels per coil	12	8	8	12	12	32	8	24	12	12	12	12	8	12	12
FoV, mm	212	256	256	192	212	212	256 × 192	212	212	212	212	212	192	212	212
Matrix	64 × 64	64 × 64	64 × 64	64 × 64	64 × 64	64 × 64	64 × 48	64 × 64	64 × 64	64 × 64	64 × 64	64 × 64	64 × 64	64 × 64	64 × 64
No. of slices	40	32	32	38	40	40	30	40	39	39	39 or 40	39 or 40	39	40	41
No. of volumes	244	143	143	107	240	240	177	240	240	240	240	240	200	240	240
In-plane resolution, mm	3.3 × 3.3	4.0 × 4.0	4.0 × 4.0	3.0 × 3.0	3.3 × 3.3	3.3125 × 3.3125	4.0 × 4.0	3.3 × 3.3	3.3 × 3.3	3.3 × 3.3	3.3 × 3.3	3.3 × 3.3	3.0 × 3.0	3.3 × 3.3	3.3 × 3.3
Slice thickness, mm	3.2	4	4	3	3.2	3.2	4.0	3.2	3.2	3.2	3.2	3.2	3.0	3.2	3.2
Slice gap, mm	0.8	0	0	0	0.8	0.8	0	0.8	0.8	0.8	0.8	0.8	0	0.8	0.8
TR, ms	2,500	2,000	2,000	2,700	2,500	2,500	2,000	2,500	2,500	2,500	2,500	2,500	2,000	2,500	2,500
TE, ms	30	27	27	31	30	30	30	30	30	30	30	30	30	30	30
Total scan time, min:s	10:17	4:46	4:46	4:49	10:00	10:00	6:00	10:00	10:00	10:00	10:00	10:00	6:40	10:00	10:00
Flip angle, deg	80	90	90	90	80	80	90	80	80	80	80	80	80	80	80
Slice acquisition order	Ascending	Ascending	Ascending	Ascending	Ascending	Ascending	Ascending	Ascending	Ascending	Ascending	Ascending	Ascending	Ascending	Ascending	Ascending
Phase encoding	PA	PA	AP	AP	AP	PA	AP	PA	PA	PA	PA	PA	PA	PA	AP
Eyes closed/fixated	Fixated	Fixated	Fixated	Fixated	Fixated	Fixated	Fixated	Fixated	Fixated	Fixated	Fixated	Fixated	Closed	Fixated	Fixated

FoV: Field of view; TR, repetition time; TE, echo time; AP, anterior to posterior; PA, posterior to anterior; SWA, Showa university; HUH, Hiroshima University Hospital; HRC, Hiroshima Rehabilitation Center; HKH, Hiroshima Kajikawa Hospital; COI, Hiroshima COI; KUT, Kyoto University TimTrio; KTT, Kyoto University Trio; UTO, University of Tokyo Hospital; ATV, ATR Verio; ATT, ATR Trio; KPU, Kyoto Prefectural University of Medicine; OSU, Osaka university; NKN, Nishinomiya Kyouritsu Hospita; CIN, CiNet.

**Table 6:** Imaging protocols for structural MRI in the SRPBS Multi-disorder Connectivity and MRI Datasets.

Site	SWA	HUH	HRC	HKH	COI	KUT	KTT	UTO	ATV	ATV	ATT	ATT	KPU	OSU, NKN	CIN
MRI scanner	*Siemens*	*GE*	*GE*	*Siemens*	*Siemens*	*Siemens*	*Siemens*	*GE*	*Siemens*	*Siemens*	*Siemens*	*Siemens*	*Philips*	*Siemens*	*Siemens*
	*Verio*	*Signa HDxt*	*Signa HDxt*	*Spectra*	*Verio.Dot*	*TimTrio*	*Trio*	*MR750w*	*Verio*	*Verio*	*TimTrio*	*TimTrio*	*Achieva*	*TimTrio*	*TimTrio*
FoV, mm	256	256	256	256	256	225 × 240	225 × 240	240	256	256	256	256	256	256	256
Matrix	256 × 256	256 × 256	256 × 256	256 × 256	256 × 256	240 × 256	240 × 256	256 × 256	256 × 256	256 × 256	256 × 256	256 × 256	256 × 256	256 × 256	256 × 256
Voxel size, mm^3^	1 × 1 × 1	1 × 1 × 1	1 × 1 × 1	1 × 1 × 1	1 × 1 × 1	0.9375 × 0.9375 × 1.0	0.9375 × 0.9375 × 1.0	1 × 1 × 1.2	1 × 1 × 1	1 × 1 × 1	1 × 1 × 1	1 × 1 × 1	1 × 1 × 1	1 × 1 × 1	1 × 1 × 1
TR, ms	2300	6812	6812	1900	2300	2000	2000	7.7	2250	2300	2250	2300	7.1	2300	1900
TE, ms	2.98	1896	1896	2.38	2.98	3.4	4.38	3.1	3.06	2.98	3.06	2.98	3.3	2.98	2.48
TI, ms	900	450	450	900	900	990	990	400	900	900	900	900	502	900	900
Flip angle, deg	9	20	20	10	9	8	8	11	9	9	9	9	10	9	9

FoV: Field of view; TR, repetition time; TE, echo time; TI, inversion time; SWA, Showa university; HUH, Hiroshima University Hospital; HRC, Hiroshima Rehabilitation Center; HKH, Hiroshima Kajikawa Hospital; COI, Hiroshima COI; KUT, Kyoto University TimTrio; KTT, Kyoto University Trio; UTO, University of Tokyo Hospital; ATV, ATR Verio; ATT, ATR Trio; KPU, Kyoto Prefectural University of Medicine; OSU, Osaka university; NKN, Nishinomiya Kyouritsu Hospita; CIN, CiNet.

Data for the Traveling Subject Dataset (Dataset 4) were acquired at sites included in the SRPBS multi-disorder database, as well as three additional sites: Kyoto University, which uses Siemens Skyra scanners, and Yaesu Clinics 1 and 2, which use Philips Achieva scanners. Each participant underwent three 10-min rs-fMRI sessions at each of nine sites, two 10-min sessions at each of two sites (Hiroshima Kajikawa Hospital and Hiroshima University Hospital), and five cycles (morning, afternoon, the following day, the following week, and the following month) consisting of three 10-min sessions at a single site (Advanced Telecommunications Research Institute [ATT]). One participant underwent four, rather than five sessions at the ATT site because of poor physical condition. Thus, a total of 143 sessions were conducted. There were two phase-encoding directions (PA and AP), three MRI manufacturers (Siemens, GE, and Philips), four channels per coil (8, 12, 24, and 32), and seven scanner types (TimTrio, Verio, Skyra, Spectra, MR750W, SignaHDxt, and Achieva). Detailed imaging parameters used at each site for fMRI and T1-weighted (T1w) structural MRI are summarized in Tables 7 and 8, respectively. Please refer to our previous paper^17^ for detailed methods.

**Table 7:** Imaging protocols for rs-fMRI in the SRPBS Traveling Subject MRI Dataset.

Site	ATT	ATV	COI	HUH	HKH	KPU	SWA	KUT	KUS	UTO	YC1	YC2
MRI scanner	*Siemens*	*Siemens*	*Siemens*	*GE*	*Siemens*	*Philips*	*Siemens*	*Siemens*	*Siemens*	*GE*	*Philips*	*Philips*
	*TimTrio*	*Verio*	*Verio*	*Signa HDxt*	*Spectra*	*Achieva*	*Verio*	*TimTrio*	*Skyra*	*MR750W*	*Achieva*	*Achieva*
Magnetic field strength	3.0 T	3.0 T	3.0 T	3.0 T	3.0 T	3.0 T	3.0 T	3.0 T	3.0 T	3.0 T	3.0 T	3.0 T
No. of channels per coil	12	12	12	8	12	8	12	32	32	24	8	8
FoV, mm	212 × 212	212 × 212	212 × 212	212 × 212	212 × 212	212 × 212	212 × 212	212 × 212	212 × 212	212 × 212	212 × 212	212 × 212
Matrix	64 × 64	64 × 64	64 × 64	64 × 64	64 × 64	64 × 64	64 × 64	64 × 64	64 × 64	64 × 64	64 × 64	64 × 64
No. of slices	40	39	40	35	35	40	40	40	40	40	40	40
No. of volumes	240	240	240	240	240	240	240	240	240	240	240	240
In-plane resolution, mm	3.3125 × 3.3125	3.3125 × 3.3125	3.3125 × 3.3125	3.3125 × 3.3125	3.3125 × 3.3125	3.3125 × 3.3125	3.3125 × 3.3125	3.3125 × 3.3125	3.3125 × 3.3125	3.3125 × 3.3125	3.3125 × 3.3125	3.3125 × 3.3125
Slice thickness, mm	3.2	3.2	3.2	3.2	3.2	3.2	3.2	3.2	3.2	3.2	3.2	3.2
Slice gap, mm	0.8	0.8	0.8	0.8	0.8	0.8	0.8	0.8	0.8	0.8	0.8	0.8
TR, ms	2,500	2,500	2,500	2,500	2,500	2,500	2,500	2,500	2,500	2,500	2,500	2,500
TE, ms	30	30	30	30	30	30	30	30	30	30	30	30
Total scan time, min:s	10:00	10:00	10:00	10:00	10:00	10:00	10:00	10:00	10:00	10:00	10:00	10:00
Flip angle, deg	80	80	80	80	80	80	80	80	80	80	80	80
Slice acquisition order	Ascending	Ascending	Ascending	Ascending	Ascending	Ascending	Ascending	Ascending	Ascending	Ascending	Ascending	Ascending
Phase encoding	PA	PA	AP	PA	PA	AP	PA	PA	AP	PA	AP	AP
Eyes closed/fixated	Fixated	Fixated	Fixated	Fixated	Fixated	Fixated	Fixated	Fixated	Fixated	Fixated	Fixated	Fixated
Field map	✓	✓	✓	—	—	✓	✓	✓	✓	✓	✓	✓

FoV: Field of view; TR, repetition time; TE, echo time; AP, anterior to posterior; PA, posterior to anterior.

ATT, ATR Trio; ATV, ATR Verio; COI, Hiroshima COI; HUH, Hiroshima University Hospital; HKH, Hiroshima Kajikawa Hospital; KPU, Kyoto Prefectural University of Medicine; SWA, Showa university; KUT, Kyoto University TimTrio; KUS, Kyoto University Skyra; UTO, University of Tokyo Hospital; YC1: Yaesu Clinic 1; YC2: Yaesu Clinic 2.

**Table 8:** Imaging protocols for structural fMRI in the SRPBS Traveling Subject MRI Dataset.

Site	ATT/ATV	COI	HUH	HKH	KPM	SWA	KUT	KUS	UTO	UTO	YC1	YC2
MRI scanner	*Siemens*	*Siemens*	*GE*	*Siemens*	*Philips*	*Siemens*	*Siemens*	*Siemens*	*GE*	*GE*	*Philips*	*Philips*
	*TimTrio*	*Verio.Dot*	*Signa HDxt*	*Spectra*	*Achieva*	*Verio*	*TimTrio*	*Skyra*	*MR750w*	*MR750w*	*Achieva*	*Achieva*
FoV, mm	256	240 × 256	256 × 256	240 × 240	256 × 256	256 × 256	225 × 240 (Axial)^*2^	232 × 256	256 × 256	256 × 256	256 × 256	256 × 256
Matrix	256 × 256	240 × 256	256 × 256	320 × 320	256 × 256	256 × 256	240 × 256	232 × 256	256 × 256	256 × 256	256 × 256	256 × 256
Voxel size, mm^3^	1× 1× 1	1× 1× 1	1× 1× 1	0.8× 0.75× 0.75	1× 1× 1	1× 1× 1	0.9375× 0.9375× 1.0	1× 1× 1	1.0000× 1.0156× 1.0156	1.2000× 1.0156× 1.0156	1× 1× 1	1× 1× 1
TR, ms	2300	2300	6788	1900	7.1	2300	2000	2300	7.7	7.7	6.99	7.01
TE, ms	2.98	2.98	1.928	2.38	3.31	2.98	3.4	2.98	3.1	3.1	3.176	3.155
TI, ms	900	900	450	900	502.6	900	990	900	400	400	—	—
Flip angle, deg	9	9	20	10	10	9	8	9	11	11	9	9

FoV: Field of view; TR, repetition time; TE, echo time; TI, inversion time; AP, anterior to posterior; PA, posterior to anterior. ATT, ATR Trio; ATV, ATR Verio; COI, Hiroshima COI; HUH, Hiroshima University Hospital; HKH, Hiroshima Kajikawa Hospital; KPU, Kyoto Prefectural University of Medicine; SWA, Showa university; KUT, Kyoto University TimTrio; KUS, Kyoto University Skyra; UTO, University of Tokyo Hospital; YC1: Yaesu Clinic 1; YC2: Yaesu Clinic 2.

### Preprocessing and calculation of the resting-state functional connectivity matrix (Dataset 1)

rs-fMRI data were preprocessed using SPM8 implemented in MATLAB (R2016b; Mathworks, Natick, MA). The first 10 s of data were discarded to allow for T1 equilibration. Preprocessing steps included slice-timing correction, realignment, co-registration, segmentation of T1-weighted structural images, normalization to Montreal Neurological Institute space, and spatial smoothing with an isotropic Gaussian kernel of 6 mm full width at half maximum (FWHM). For analysis of connectivity matrices, regions of interest (ROIs) were delineated according to 140 regions covering the entire brain, defined anatomically by the digital atlas of the Brainvisa Sulci Atlas and three subregions of the cerebellum (the left and right cerebellum, and the vermis). Blood oxygen level-dependent (BOLD) signal time courses were extracted from these 140 ROIs. A bandpass filter (transmission range, 0.008–0.1 Hz) was applied to these sets of time courses before the following regression procedure. Filtered time courses were linearly regressed on temporal fluctuations of the white matter, the cerebrospinal fluid, and the entire brain, as well as six head motion parameters. Here, fluctuation in each tissue class was determined from the average time course of the voxels within a mask created by the segmentation procedure of the T1 image. The mask for the white matter was eroded by one voxel to consider the partial volume effect. These extracted time courses were bandpass filtered (transmission range, 0.008–0.1 Hz) before linear regression, as was done for regional time courses. Then, for each individual, a matrix of 9,730 functional connections between 140 ROIs was calculated by exhaustively evaluating pair-wise temporal Pearson correlations of BOLD signal time courses, while discarding any flagged frames in the previous procedure (scrubbing). We calculated the framewise displacement (FD) and removed volumes with FD >0.5 mm, as proposed in a previous study^25^. Demographic information of Dataset 1 includes the rate of excluded volumes and the number of excluded volumes for each participant. For details about the entire procedure to calculate the matrix, see Yahata *et al*.^13^.

### Face-masking of structural MRI data (Datasets 2 and 3)

**Fig. 2:**
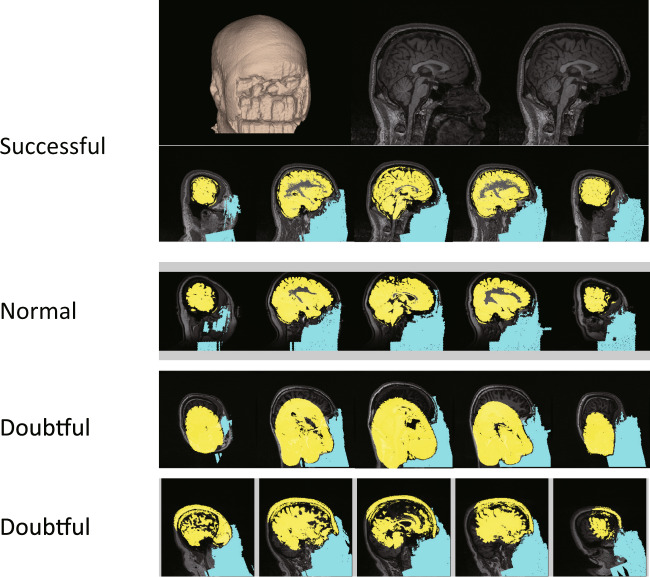
Face-masking process. We removed signals of volumes covering the facial surface. This process generates a report file of the face-masking results for each participant, containing a 3D reconstruction of the surface of defaced T1w images.

To prevent identification of individual participants via reconstruction of the facial surface from structural MRI data, we performed a face-masking calculation. We removed signals of volumes covering the facial surface. The code, which is available on our GitHub project (https://github.com/bicr-resource/deface), removes the subject’s face from the MRI structure image (NIfTI format). The code is written in MATLAB and internally uses SPM8 and *mri_deface*. Finally, a report file of face-masking results is generated for each participant (Fig. 2). The report contains a 3D reconstruction of the surface of defaced T1w images, two sagittal slices at x = 0 before and after face-masking, five sagittal slices of the detected brain (colored yellow), and removed voxels (colored by cyan) at different x-coordinates (x = {−60, −30, 0, 30, 60} (mm) in naive space). We used this report to assess the quality of the face-masking process (see Technical Validation).

## Data Records

All datasets are available on Synapse (Synapse ID: syn22317076)^18–21^. A summary of data records for all datasets is presented in Table 9. Full datasets are also available on the DecNef Project Brain Data Repository website (https://bicr-resource.atr.jp/decnefpro/). Please see Usage Notes section for more information on requirements to access datasets.

**Table 9:** Summary of data records for each dataset.

Name	Dataset 1	Dataset 2	Dataset 3	Dataset 4
	SRPBS Multi-disorder Connectivity Dataset	SRPBS Multi-disorder MRI Dataset		SRPBS Traveling Subject MRI Dataset
**Synapse ID**	syn22317076			
**Availability**	Application and approval required	Application and approval required	Registration required	Registration required
**DOI**	10.7303/syn22317078	10.7303/syn22317079	10.7303/syn22317081	10.7303/syn22317082
**Summary of contents**	1) Resting state functional connectivity matrix (.mat file) 2) Participant demographics 3) README (with MRI protocols)	1) Brain imaging dataset (NIFTI) – rsfmri, T1w, fieldmap 2) Participant demographics 3) README 4) MRI protocols 5) QC results	1) Brain imaging dataset (NIFTI) – rsfmri, T1w, fieldmap 2) Participant demographics 3) README 4) MRI protocols 5) QC results	1) Brain imaging dataset (NIFTI with BIDS) – rsfmri, T1w, fieldmap 2) Participant demographica 3) README 4) MRI protocols 5) QC results
**Details of contents**				
**MRI**	Each consortium site generated one.mat file containing the values of functional connectivity for each participant [number of participants × number of functional connectivities]	Each of 1,627 subject folders contains - rsfmri (resting-state fMRI EPI images) - t1 (T1-weighted structural image) - fmap (field-map, optional)	Each of 1,410 subject folders contains - rsfmri (resting-state fMRI EPI images) - t1 (T1-weighted structural image) - fmap (field-map, optional)	Each of 143 session folders contain - func (resting-state fMRI EPI images) - anat (T1-weighted structural image) - fmap (field-map, optional)
**Demographics**	subject ID, age, sex, handedness, diagnosis, auxiliary demographic information, rate of excluded volume, number of excluded volume	subject ID, age, sex, handedness, diagnosis, auxiliary demographic information	subject ID, age, sex, handedness, diagnosis, auxiliary demographic information	BIDS ID, subject ID, site, number of repetitions, phase encoding, MRI manufacturer, number of coils, scanner, session id, sex, age

Dataset 1 comprises the resting-state functional connectivity matrix data (.mat) with the number of (participants) × (the number of functional connectivities = 9,730) calculated for each site;^18^ participant demographic information (.csv); and readme file with MRI parameters (.txt) of each site. See Table 2 for the number of participants for each site.

Dataset 2 comprises brain imaging data from 1627 participants in NIFTI format^19^. The “data” folder includes 1627 folders labeled with participant ID numbers (ex. sub-0001). Each participant folder contains folders labeled “rsmri,” which contain rs-fMRI EPI images with the number of volumes (.nii); “t1,” which contain defaced, T1-weighted structural images (.nii), and optionally “fmap,” which contain field-map images (.nii). Dataset 2 also contains demographic information (“participants_diagscore.xlsx”), MRI parameters (“MRI_protocols.xlsx”), quality control (QC) results (“group_T1w.tsv” and “group_bold.tsv”; see Technical Validation), and a text file (“README.txt”) explaining the abbreviations that appear in other files.

Dataset 3 comprises brain imaging data in NIFTI format from 1410 participants, including those in Dataset 2 who consented to having their data shared publicly^20^. Thus, the data structure is the same as that of Dataset 2.

Dataset 4 comprises brain imaging data from 143 sessions in NIFTI format^21^. Dataset 4 is a BIDS-validated dataset^26^. The “sourcedata” folder contains 143 folders labeled with participant ID numbers (ex. sub-001). Each participant folder contains folders labeled “func,” which contain rs-fMRI EPI images (.nii.gz). “anat” contains defaced T1-weighted structural images (.nii.gz). “fmap” contains field-map images (.nii.gz). Dataset 2 also contains “participants.tsv,” which includes demographic data (BIDS ID, subject ID, site, number of repetitions, phase encoding, MRI manufacture, coil, scanner, session id, sex, age). “dataset_description.json” includes information about the dataset, QC results (“group_T1w.tsv” and “group_bold.tsv”; see Technical Validation), and a text file (“README.txt”) summarizing the dataset.

In all datasets, demographic information includes unique participant ID numbers, sex, and age. Datasets 1–3 additionally include participant handedness, diagnosis, and auxiliary demographic information.

All datasets include information about imaging parameters (Tables 5–7): the MRI scanner model, magnetic field strength, number of channels per coil, FoV (mm), matrix, number of slices, number of volumes, in-plane resolution (mm), slice thickness (mm), slice gap (mm), TR (ms), echo time (ms), inversion time (ms; for T1w only), total scan time (min:s), flip angle (deg), slice acquisition order, phase encoding, eyes closed/fixated.

## Technical Validation

### Quality assessment of fMRI and structural MRI data (Datasets 2–4)

**Fig. 3:**
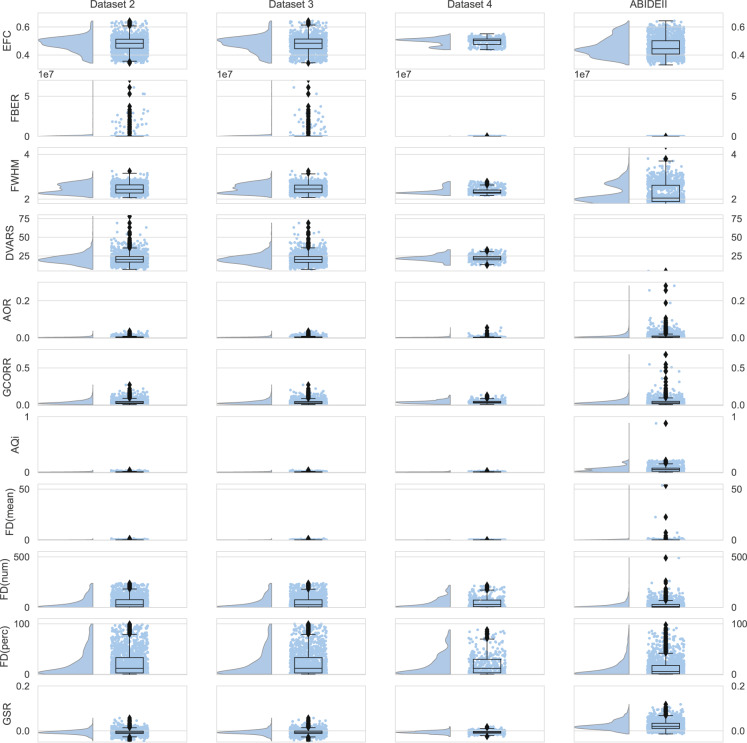
Quality metrics of rs-fMRI. Quality metrics of rs-fMRI of Datasets 2–4 and the open dataset (ABIDEII, http://fcon_1000.projects.nitrc.org/indi/abide/abide_II.html). See the “Technical Validation” section for definitions of all abbreviations.

**Fig. 4:**
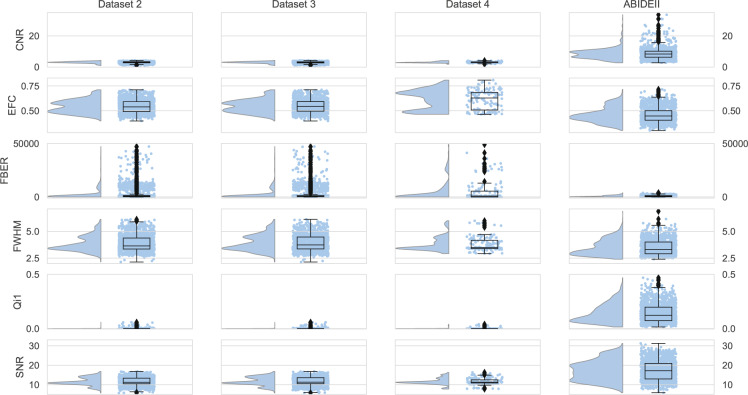
Quality metrics of T1w. Quality metrics of T1w structural MRI of Datasets 2–4 and the open dataset. See the “Technical Validation” section for definitions for all abbreviations.

For Datasets 2–4, consistent with the MRI data, we calculated quality metrics by applying MRIQC (Esteban *et al*., 2017). We plotted the spatial and temporal measures listed below for Datasets 2–4 and the open dataset (ABIDEII, http://fcon_1000.projects.nitrc.org/indi/abide/abide_II.html) for comparisons (Figs. 3 and 4). For detailed descriptions of those metrics and related references, please visit the PCP Quality Assessment Protocol website (http://preprocessed-connectomes-project.org/quality-assessment-protocol/). We used *Raincloud plot* in the *ptitprince* python package (https://github.com/RainCloudPlots/RainCloudPlots)^27^ for the strip plot, box plot, and violin plot for each dataset.

**Measures of rs-fMRI (Fig. 3)**

**Entropy focus criterion (EFC):** The Shannon entropy of voxel intensities proportional to the maximum possible entropy for an image of similar size. Indicates ghosting and head motion-induced blurring. Lower values are better.

**Foreground-to-background energy ratio (FBER):** The variance of voxels inside the brain divided by the variance of voxels outside the brain. Higher values are better.

**Voxel smoothness (FWHM):** The FWHM of the spatial distribution of image intensity values in voxel units. Lower values are better.

**Standardized DVARS (DVARS):** The average change in mean intensity between each pair of fMRI volumes in a series scaled to enable comparisons among multiple scanning protocols. Lower values are better.

**Outlier detection (AOR):** The mean count of outliers found in each volume using the 3dToutcount command from AFNI. Lower values are better.

**Global correlation (GCORR):** The average correlation of all pairs of voxel time series in the brain. Illustrates differences among data due to motion/physiological noise/imaging artifacts (such as signal bleeding). Values closer to zero are better.

**Median distance index (AQi):** The mean distance (1 – Spearman’s rho) between each time point’s volume and the median volume using AFNI’s 3dTqual command. Lower values are better.

**Ghost-to-signal Ratio (GSR):** A measure of the mean signal in areas of the image that are prone to ghosting based on the phase encoding direction. Lower values are better.

**Mean RMSD (FD [mean]):** A measure of head motion, which compares the motion between the current and previous volumes. This is calculated by summing the absolute value of displacement changes in the x, y, and z directions and the rotational changes about those three axes. Rotational changes are given distance values based on the changes across the surface of a sphere with a radius of 80 mm. Lower values are better.

**Number of volumes with a framewise displacement greater than 0.2 mm (FD [num]):** Lower values are better.

**Percent of volumes with a framewise displacement greater than 0.2 mm (FD [perc]):** Lower values are better.

**Measures of anatomical MRI (T1w) (Fig. 4)**

**Contrast-to-noise ratio (CNR):** The mean of gray matter intensity values minus the mean of white matter intensity values divided by the standard deviation of values outside the brain. Higher values are better.

**Entropy focus criterion (EFC):** The Shannon entropy of voxel intensities proportional to the maximum possible entropy for a similarly sized image. Indicates ghosting and head motion-induced blurring. Lower values are better.

**Foreground-to-background energy ratio (FBER):** The variance of voxels inside the brain divided by the variance of voxels outside the brain. Higher values are better.

**Voxel smoothness (FWHM):** The FWHM of the spatial distribution of the image intensity values in voxel units. Lower values are better.

**Percent artifact voxels (Qi1):** The ratio of voxels outside the brain with artifacts to the total number of voxels outside the brain. Lower values are better.

**Signal-to-noise ratio (SNR):** The mean intensity in the gray matter divided by the standard deviation of values outside the brain. Higher values are better.

### Quality assessment of structural MRI data face-masking (Datasets 2 and 3)

We manually evaluated the quality of face-masking processing. Three people (one neuroimaging researcher and two clinical psychologists) independently evaluated the accuracy of face-masking by visually checking the structural MRI data. In the evaluation, “1” is successful, “2” is normal, and “3” is doubtful (e.g., part of the brain is missing or part of the face is present). See example images for “successful,” “normal,” and “doubtful” in Fig. 2. Numbers of participants evaluated as “successful,” “normal,” and “doubtful” were 1561, 60, and 6 in Dataset 2 and 1344, 60, and 6 in Dataset 3, respectively.

## Usage Notes

Interested parties can visit the Synapse project site (Synapse ID: syn22317076) or the DecNef Project Brain Data Repository site (https://bicr.atr.jp/decnefpro/data) to apply for access to datasets.

Before submitting access requests, applicants should read the privacy policy for each dataset. Datasets may not be used for commercial purposes. Datasets may not be copied or redistributed. If applicants publish manuscripts using these datasets, applicants agree to acknowledge the DecNef Project Brain Data Repository as the data source and to include language similar to the following: “Data used in the preparation of this work were obtained from the DecNef Project Brain Data Repository (https://bicr-resource.atr.jp/srpbsopen/), collected as part of the Japanese Strategic Research Program for the Promotion of Brain Science (SRPBS) supported by the Japanese Advanced Research and Development Programs for Medical Innovation (AMED).” Note that we periodically collect the number of applicants and the amount of data downloaded from the database for reports to the DecNef Project, but not private information. Note that parties wishing to use these data must review and agree to these terms, including those who access shared copies of the data. In the event that data need to be shared with collaborators, those persons must also register with the respective data repositories and agree to all terms. Each dataset has a different application process, as described below.

### From Synapse

Applicants need a valid Synapse account. Access is controlled by separate conditions for use, so please check the Synapse project wiki for the terms of use for each dataset.

1. **The SRPBS Multi-disorder Connectivity Dataset**^18^
  Applicants download the “Application Form for Data Usage” from the Synapse project site (10.7303/syn22317078), and email the completed form to decnef-db-admin@atr.jp. We will add download privileges to the applicant’s Synapse account.
2. **The SRPBS Multi-disorder MRI Dataset (restricted)**^19^
  Applicants download the “Application Form for Data Usage” from the Synapse project site (10.7303/syn22317079), and **email** the completed form to decnef-db-admin@atr.jp. We will add download privileges to the applicant’s Synapse account.
3. **The SRPBS Multi-disorder MRI Dataset (unrestricted)**^20^
  Applicants must have a valid Synapse account to access the Synapse project site (10.7303/syn22317081).
4. The SRPBS Traveling Subject MRI Dataset^21^

Applicants must have a valid Synapse account to access the Synapse project site (10.7303/syn22317082).

From the DecNef Project Brain Data Repository Site:

1. **The SRPBS Multi-disorder Connectivity Dataset**
  Applicants download the “Application Form for Data Usage” from the repository site (https://bicr-resource.atr.jp/srpbsfc), and **email** the completed form to decnef-db-admin@atr.jp. Login information and instructions for accessing the database and downloading data to the applicant’s computer will be sent via e-mail.
2. **The SRPBS Multi-disorder MRI Dataset (restricted)**
  Applicants download the “Application Form for Data Usage” from the repository site (https://bicr-resource.atr.jp/bicr_add/jsp/appForm.jsp), and **upload** the completed form and an image file of applicant’s signature separately. Login information and instructions for accessing the database and downloading data to applicant’s computer will be sent via e-mail.
3. **The SRPBS Multi-disorder MRI Dataset (unrestricted)**
  Applicants complete and submit **the online registration form** (https://bicr-resource.atr.jp/accounts/create_srpbsopen). The user account and login information will be issued automatically via email. Applicants can access data by logging into their accounts.
4. **The SRPBS Traveling Subject MRI Dataset**

Applicants complete and submit **the online registration form** (https://bicr-resource.atr.jp/accounts/create_srpbsts). The user account and login information will be issued automatically via email. Applicants can access data by logging in to their accounts.

We set up contact information (decnef-db-admin@atr.jp) for inquiries about the usage of the datasets.

## Data Availability

The face-masking code is available on our GitHub project. (https://github.com/bicr-resource/deface).
